# A Domestic Revolution

**Published:** 1869-10

**Authors:** 


### A DOMESTIC REVOLUTION.

There are thousands of lady housekeepers in New York, and thousands more in other cities, whose domestic happiness is daily poisoned, and their lives made miserable, by the cooks in the kitchen and the maids in the parlor. There will be a speedy change in all this. The Chinese will flock to our shores by millions. We saw a very handsome man-servant doing duty in a Fifth Avenue mansion the other day. When from the middle classes, the Chinese are a patient, docile, imitative race. A patience and a docility which have become a part of their nature, from the grinding oppressions and despotisms of centuries, are qualities very essential as servants to Americans, because we are an impatient, inconsiderate, and hasty race; and when a passionate mistress gets hold of a passionate maid, then Greek meets Greek, and there is a general flare up. The Chinese are teachable. Once telling is enough. You need not even tell, need not waste breath, but just show him once how to do a thing, and he will never forget it the longest day he lives; he will follow the thing up literally for the remainder of his natural life. You tell Bridget to do a particular thing in a particular way; yes'm is the ready response, and she goes right off to do it as she had done it before. You may tell her a million times; a million times she will say yes'm and no'm. Where is the lady housekeeper who has not ground her teeth in rage, or bit her lips in her irrepressibility a thousand times at this very doggedness? Some time ago a lady took the pains to show a Chinese servant how to mix up eggs with flour for batter; she took up five eggs, but the fifth not being good, she threw it away. Some months later, happening to go into the kitchen while the faithful John was preparing batter, she observed that he threw the last egg away, although it was perfectly good. On inquiry, it was found that he had been throwing a good egg away, the last of the lot, every day for six months. He did as the mistress did. What an immeasurable relief it would be to any of our housekeepers to know that for ever hereafter there would be no occasion to tell or show a servant the same thing twice. We think such servants will command a premium of wages. To have patient, gentle, docile, faithful servants what a household millennium it will be! Another advantage is, they are willing to work for very low wages.

Bridget is the most despotic and exacting autocrat in Christendom to-day. But the sceptre is broken, the glory has departed, and our wives are free. Hurrah for the grand Pacific railroad! Every body ought to take stock in it, especially as it pays good and gold interest.

